# Items

**Published:** 1875-07

**Authors:** 


Illinois State Medical Society. Dr. Thomas D. Washburn, of Hillsboro, who has occasionally contributed articles to our pages, was elected president of this society at its last annual meeting in May. The honor is worthily bestowed. ——Intercurrent Cerebral Meningitis. Dr. Walter Hay describes in the *Chicago Medical Journal* for July, a case of cerebral meningitis occurring in connection with a case of measles which presented certain features which he does not find described by authors. Under appropriate treatment the case convalescent on the eighth day of the disease.——Honors to Dr. Tilt. Dr. Tilt's *Handbook of Uterine Therapeutics* and his work upon *Change of Life* have been translated into Italian. The Italian government have made him a Knight of the

Crown of Italy.——LIEBIG'S SUCCESSOR. The successor of this eminent teacher of chemistry in the University of Munich, is to be Prof. Baeyer of Strasburg.——THE BROMPTON CONSUMPTION HOSPITAL. By a recent bequest from a lady this famous hospital has been made the recipient of one half million dollars in gold. Its present income is nearly ninety thousand dollars. During the last year fifteen thousand five hundred patients were treated in the out-door department.——MEASLES IN FIJI ISLANDS. Since the annexation of these Islands to British domain the measles have been introduced and are nearly depopulating the islands. The epidemic rages with unusual virulance among the natives, eight to ten deaths occurring in a day. The families of the white residents have been attacked but not with more than the usual severity.——NEW MEDICO—LEGAL WORK. Dr. M. Gonzales Echeverria, late of New York, is soon to published in London a work on the medical and legal aspects of epilepsy.——HOSPITAL AT FLORENCE. In Florence where so much has been said against the vivisections of Prof. Schiff, there seems to be need of a reform in the conduct of the hospital. The odor is said to be perceptible to persons passing on the street, and by good authority it is said that the most severe operations are made without the use of anæsthetics. ——RETIRED. Owing to ill health Dr. W. F. Jenks has been relieved from the editorship of the American supplement of the *Obstetrical Journal of Great Britain and Ireland.* He is succeeded by Dr. J. V. Ingham.

